# Differential effects of hemodialysis modalities on circulating neutrophil extracellular traps in children on maintenance hemodialysis: a cohort study

**DOI:** 10.1038/s41598-025-18791-4

**Published:** 2025-09-17

**Authors:** Mohammed F. Kasem, Ragia M. Said, Noha R. Mohammed, Aya M. Sultan, Eman R. Edris, Noha U. Hashem

**Affiliations:** 1 Division of Pediatric Nephrology, Department of Pediatrics, Faculty of Medicine, Ain Shams University, Cairo, Egypt; 2 Department of Clinical Pathology, Faculty of Medicine, Ain Shams University, Cairo, Egypt; 3 Egyptian Ministry of Health, Cairo, Egypt

**Keywords:** Neutrophil extracellular traps (NETs), Chronic kidney disease (CKD), Hemodialysis (HD), Low-flux hemodialysis (LFHD), High-flux hemodialysis (HFHD), Online hemodiafiltration (OL-HDF), Biomarkers, Medical research, Nephrology

## Abstract

Flow of blood through the extracorporeal circuit during conventional hemodialysis (HD) can induce neutrophil activation, leading to inflammatory and cytokine responses. So, we evaluated the effect of modality and membrane performance of dialysis session in children on maintenance HD on neutrophil activation by measuring the level of circulating neutrophil extracellular traps (NETs). Twenty-six incident children stable on LFHD for at least 3 months were recruited along with 26 age- and sex-matched healthy controls. Pre-and post-session blood samples for complete blood count (CBC), C - reactive protein (CRP), and NETs were obtained on a mid-week session. Patients were shifted to HFHD for 3 months, followed by post-dilutional OL-HDF for additional 3 months. Blood tests were repeated at the end of each interval. Post-LFHD NETs levels were significantly higher than pre-LFHD *(p = < 0.0001)* and control levels *(p < 0.0001).* Both HFHD and OL-HDF resulted in significant reduction in the post-session NETs levels in comparison to their pre-session levels *(p < 0.0001*,* p < 0.0001 respectively)*. However, OL-HDF resulted in a significant reduction of post- session NETs levels to a degree that they became comparable to control levels *(p = 0.928)*. We conclude that children on HD have high serum levels of NETs that were significantly increased by using LFHD membranes. HFHD and OL-HDF sessions led to significant reduction in NETs reflecting their possible role in reducing the dialysis related production of NETs, however OL-HDF was superior to HFHD, as it lowered post-session NETs to levels that were comparable to that of controls.

**Supplementary Information:**

The online version contains supplementary material available at 10.1038/s41598-025-18791-4.

## Introduction

Hemodialysis revolutionized the care of patients with end-stage kidney disease (ESKD) allowing the removal of metabolic toxins as blood flows through an extra-corporeal circuit^1^. However, this circuit of synthetic tubes and dialyzer membranes is not a natural design; contact of blood with synthetic materials of dialysis membranes during HD treatment triggers an inflammatory immune response resulting in activation of various protein cascade systems (complement and coagulation pathway), and activation of both endothelial and circulating cells (i.e. platelets, lymphocytes, neutrophils and erythrocytes) with release various soluble mediators (such as enzymes, pro-inflammatory cytokines, and reactive oxygen species)^2,3^. Such blood–membrane interactions have been an index for hemo(in)compatibility, or more broadly bio(in)compatibility of HD membranes^4,5^. Extracorporeal circuit hemo(in)compatibility contributes to endothelium dysfunction and increases thrombogenicity leading to the development of vascular disease , organ dysfunction-especially cardiovascular disease- and poor outcomes in CKD patients on maintenance HD^6,7^.

Although that the type and quality of dialysis membrane material are the primary determinants of such reaction, other essential key factors for such interactions include dialysis membrane surface roughness (porosity) along with dialyzer functional parameters^8^. The membrane roughness and its structural and chemical texture, along with molecular mass transfer, and ultrafiltration coefficients, may all also adversely influence dialysis-induced inflammatory response^8,9^. Also other dialysis-related factors that may enhance such immunological reaction include exposure to endotoxin from dialysis fluid, dialysate chemical composition, and silent vascular access infection^5^. So, using more biocompatible dialyzer membrane polymers, ultrapure dialysis water, and convective-based HD such as online hemodiafiltration (OL-HDF), that offers higher efficiency in uremic solute removal together with a reduced inflammatory response, may lessen such inflammatory reaction^10,11^. OL-HDF has been proposed to improve patients’ survival rates in comparison to conventional HD, provided that adequate convection volumes, and ultrapure water were used^12^.

Neutrophil extracellular trap (NET) formation, commonly referred to as NETosis, is a unique form of regulated neutrophil cell death characterized by the release of chromatin structures into the extracellular space to trap and neutralize pathogens^13^. This process involves a cascade of molecular and cellular events that disrupt nuclear and plasma membrane integrity. One of the earliest and most critical steps is chromatin decondensation, which is primarily mediated by peptidylarginine deiminase 4 (PAD4). PAD4 catalyzes the citrullination of histones, particularly histone H3, leading to a reduction in positive charge and subsequent loosening of the chromatin structure thus facilitating eventual release of nuclear content^14^. Following chromatin decondensation, the nuclear envelope must be dismantled to permit the exit of chromatin through phosphorylation of nuclear lamins. Specifically, lamin B is phosphorylated by protein kinase C alpha (PKCα), while lamin A and C are targeted by cyclin-dependent kinases 4 and 6 (CDK4/6), thus facilitating nuclear envelope destabilization, promoting its rupture, and enabling the decondensed chromatin to move toward the cytoplasm^15,16^. The final stage of NETosis involves the disruption of the plasma membrane, a process that requires remodeling of the actin cytoskeleton and membrane-associated structures allowing for the extrusion of chromatin fibers^17^. Together, these sequential molecular events—histone citrullination, nuclear envelope disintegration, and plasma membrane rupture—constitute the mechanistic basis of NETosis and highlight potential therapeutic targets for modulating neutrophil-driven inflammation in various diseases^13,18^. Recently, it has been postulated that NETosis, is an integrated marker of HD triggered immune reactions^5^. They have been identified as a major harmful component in a variety of vascular diseases associated with inflammatory processes^19^. It has been also shown that NETosis was able to induce platelet trapping and promote microvascular occlusions and profound venous thrombosis. leading to multiorgan failure^20,21^. NETs consist of meshed extracellular structures comprised of DNA, histones, with a variety of granular proteins such as proteases, elastase, and myeloperoxidase (MPO). Such NETs byproducts, such as elastase, MPO, and cell free DNA, have been reported to increase in HD patients, particularly during dialysis sessions indicating that NETs could be a significant marker of neutrophil-membrane interaction to focus on in HD patients^22^. However, this issue remains poorly investigated with lack of knowledge in literature addressing the influence of various types of HD and relative contributions of more efficient renal replacement therapies, such as OL-HDF per se on NETs imbalance phenomenon. So, the aim of this study was to determine whether HD session have any impact on the levels of circulating NETs in children on maintenance HD and any possible differences in relation to the performance of dialyzer membrane and the modality of HD session.

## Methods

### Study population

This study was conducted at Pediatric Dialysis Unit, Children’s Hospital of Ain Shams University over 6 months after approval of the Research Ethics Committee, Faculty of Medicine, Ain Shams University (FWA 000017585; FMASU: MS56/2023) and was performed according to the guidelines and Declaration of Helsinki. Twenty-six incident CKD-5d children who were stable on conventional HD using low flux membrane (LFHD) for more than 3 months and aged 16 years or less were recruited along with twenty-six age and sex matched healthy children as a control group. Patients with unsuitable vascular access blood flow, acute kidney injury, failed kidney graft, active infection, history of blood transfusion in the last month, autoimmune disease, active thrombosis, hepatitis, diabetes, malignancy, cardiomyopathy, infective endocarditis, on immunosuppressive drugs and CKD-5d on HD for less than 3 months were excluded. All patients underwent a baseline evaluation that included data regarding age, sex, cause of CKD, HD vintage in months, detailed medical history, and thorough physical examination mainly to rule out any recent or active infection, inflammation, or thrombosis.Demographics of the study including detailed patients’ and HD data are shown in (Table 1).

### Blood tests

**Fig. 1:**
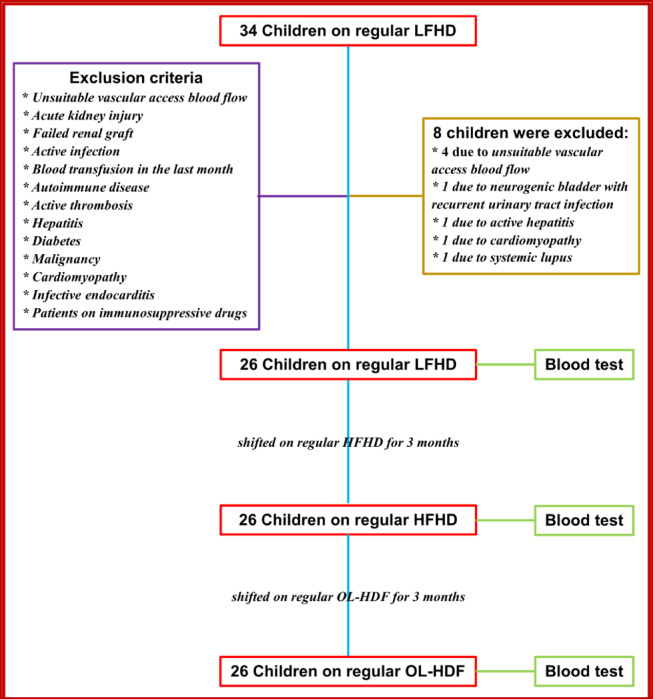
Study flowchart showing participant sequential transition through low-flux hemodialysis (LFHD), high-flux hemodialysis (HFHD) and online hemodiafiltration (OL-HDF) phases with blood testing after each.

Blood samples were collected for laboratory workup pre- and post- mid-week session for patients on LFHD. Thereafter all patients were shifted to high flux hemodialysis (HFHD) for 3 months, then shifted to post-dilution OL-HDF for another 3 months. At the end of each three-month treatment period, the same laboratory tests were repeated before and after a midweek HD session, as shown in the flow chart in (Fig. 1). Blood samples included five mL of venous blood that wereanalyzed for complete blood count (CBC), C-reactive protein (CRP), and serum NETs for patients as well as healthy controls. Pre- and post- session blood samples were obtained from a peripheral vein away from the limb having the arteriovenous fistula. Before post- session sampling, blood flow was decreased to 50 ml/minute with concomitant stoppage of the dialysate flow and replacement fluid. Blood samples were allowed to clot for 10–20 min at room temperature. To minimize residual heparin interference post-dialysis samples were allowed to clot for 45–60 min at room temperature. Samples were then centrifuged at 2000–3000 revolutions per minute (RPM) for 20 min. Two mL of blood samples were collected in ethylene diamine tetra-acetic acid (EDTA) tubes for CBC including total leukocyte count (TLC), absolute neutrophil count (ANC), absolute lymphocyte count (ALC), Hemoglobin (Hb), and Platelet (PLT) counts. Analyses were performed using *Coulter LH 750 automated haematology autoanalyzer supplied by Beckman instruments Inc. (Fullerton*,* CA 92634*,* 3100*,* USA).* One mL of serum were assayed for C-reactive protein (CRP) analysis using COBAS C6000 systems supplied by Roche Diagnostics (GmbH, Sandhofer Strasse 116, D-68305 Mannheim). Two mL of serum were used to assess NETs by measuring citrullinated histone H3 (CitH3) component using Enzyme-Linked Immunosorbent Assay (ELISA) kit (Cat. No: E4548Hu, Bioassay Technology Laboratory, Shanghai, China).

#### Objectives

To assess (1) The status of circulating NETs in children on maintenance HD (2) The post-session effect of each HD modality on circulating NETs (3) The differences between different HD modalities on post-session circulating NETs.

### Statistical analysis

Analysis of data was performed using IBM SPSS Statistics (Statistical Package for the Social Sciences), Version 26.  Shapiro-Wilk test for normality was used. Quantitative variables were described as range, mean ± standard deviation (x̄ ±SD) for normally distributed variables, and as median (Mdn), and interquartile range (IQR; the difference between 75th & 25th percentiles) for skewed data. Qualitative variables were described as numbers and percentages. To compare parametric quantitative parameters, Student t-test was applied for independent variables and paired t-Test for dependent variables. For comparison of non-parametric quantitative parameters, Mann-Whitney test was used for independent variables and Wilcoxon Signed Rank test for dependent variables. Pearson and Spearman rho test were used for studying correlations. P value < 0.05 was considered significant in all analyses.

## Results

A total of 26 incident HD patients along with 26 healthy age and sex matched controls were enrolled in this study. Patients’ mean age was 12.2 ± 2.8 years (range: 5.8–15.6 years) including 16 (61.5%) males and 10 (38.5%) females. Controls’mean age was 10.9 ± 2.7 years (range: 6.0–15.0 years) including 13 males and 13 females. All patients were clinically stable throughout the study period with comparable CRP levels with the controls (*p* > 0.05). None of our patients reported to have high CRP (i.e. CRP > 6 mg/L) at any point of time during the study. Study demographics are shown in (Table 1).

**Table 1:** Study demographics.

Parameters		Patients (*n* = 26)	Controls (*n* = 26)
Underlying kidney diagnosis (*n*) (%)		* Unknown (8) (30.77%)	-
		* CAKUT (7) (26.92%)	
		* Chronic glomerulopathy (6) (23.08%)	
		* ARPKD (2) (7.69%)	
		* Joubert syndrome (2) (7.69%)	
		* Alport syndrome (1) (3.85%)	
HD vintage (Mdn; IQR)		35.5; 32.6 months	-
Presence of hypertension		14 (53.85%)	**-**
Residual urine volume		All patients were anuric (26; 100%)	**-**
Frequency of dialysis sessions		3 sessions/week	**-**
Duration of dialysis sessions		3 h/session	**-**
*Dialyzer Material		Fresenius polysulfone for all patients (26; 100%)	**-**
*Dialyzer Surface area	LFHD	* 0.8 m^2^ (16; 61.54%), 1.0m^2^ (10; 38.46%)	**-**
	HFHD	* 0.7 m^2^ (26; 100%)	
	OLHDF	* 0.7 m^2^ (26; 100%)	
Blood lines		LFHD & HFHD: HD pediatric lines	**-**
		OL-HDF: HDF lines	
Dialysis machine		Bellco formula therapy & formula 2000 plus	**-**
Dialysis water quality		Ultrapure water & DIASAFE ^®^ filter (26; 100%)	**-**
Vascular Access		AVF in all patients (26; 100%)	**-**
Blood flow (ml/minute/m^2^) (Mdn; IQR)		* LFHD (229.7; 31.45)	**-**
		* HFHD (231.7; 28.30)	
		* OL-HDF (235.2; 24.60)	
Source of dialysis fluid		Central Dialysis Fluid Delivery System (CDDS)	**-**
Dialysate flow		500 ml/minute in all patients (26; 100%)	**-**
Dialysis bath composition Standard for all patients (26; 100%)		Sodium = 138 mmol/L, Potassium = 2 mmol/L, Calcium = 1.75 mmol/L, Magnesium = 0.5 mmol/L, Chloride = 106.5 mmol/L, Bicarbonate = 32 mmol/L	**-**
Anticoagulant		Unfractionated heparin in all patients (26; 100%)	**-**
Convection volume (Mdn; IQR)		13.24; 2.49 L/m^2^	**-**
CRP (mg/L) (Mdn; IQR)		* LFHD (1.3; 2.7)	1.4; 0.06
		* HFHD (0.9; 1.2)	
		* OL-HDF (1.1; 1.7)	
BUN (mg/dL) (Mdn; IQR)		* LFHD (72.9; 17.3)	9.82; 1.86
		* HFHD (70.7; 16.9)	
		* OL-HDF (65.4; 16.3)	
Creatinine (mg/dL) (Mdn; IQR)		* LFHD (5.51; 1.98)	0.47; 0.07
		* HFHD (5.30; 1.70)	
		* OL-HDF (4.69; 2.00)	
spKt/V (Mdn; IQR)		* LFHD (1.3; 0.1)	**-**
		* HFHD (1.35; 0.2)	
		* OL-HDF (1.43; 0.18)	

CAKUT, congenital anomalies of kidney & urinary tract; HD, hemodialysis; LFHD, low flux hemodialysis, HFHD, high flux hemodialysis; OL-HDF, online hemodiafiltration; Mdn, median; IQR, interquartile range; CRP, C-reactive protein; BUN, blood urea nitrogen.

**Fig. 2:**
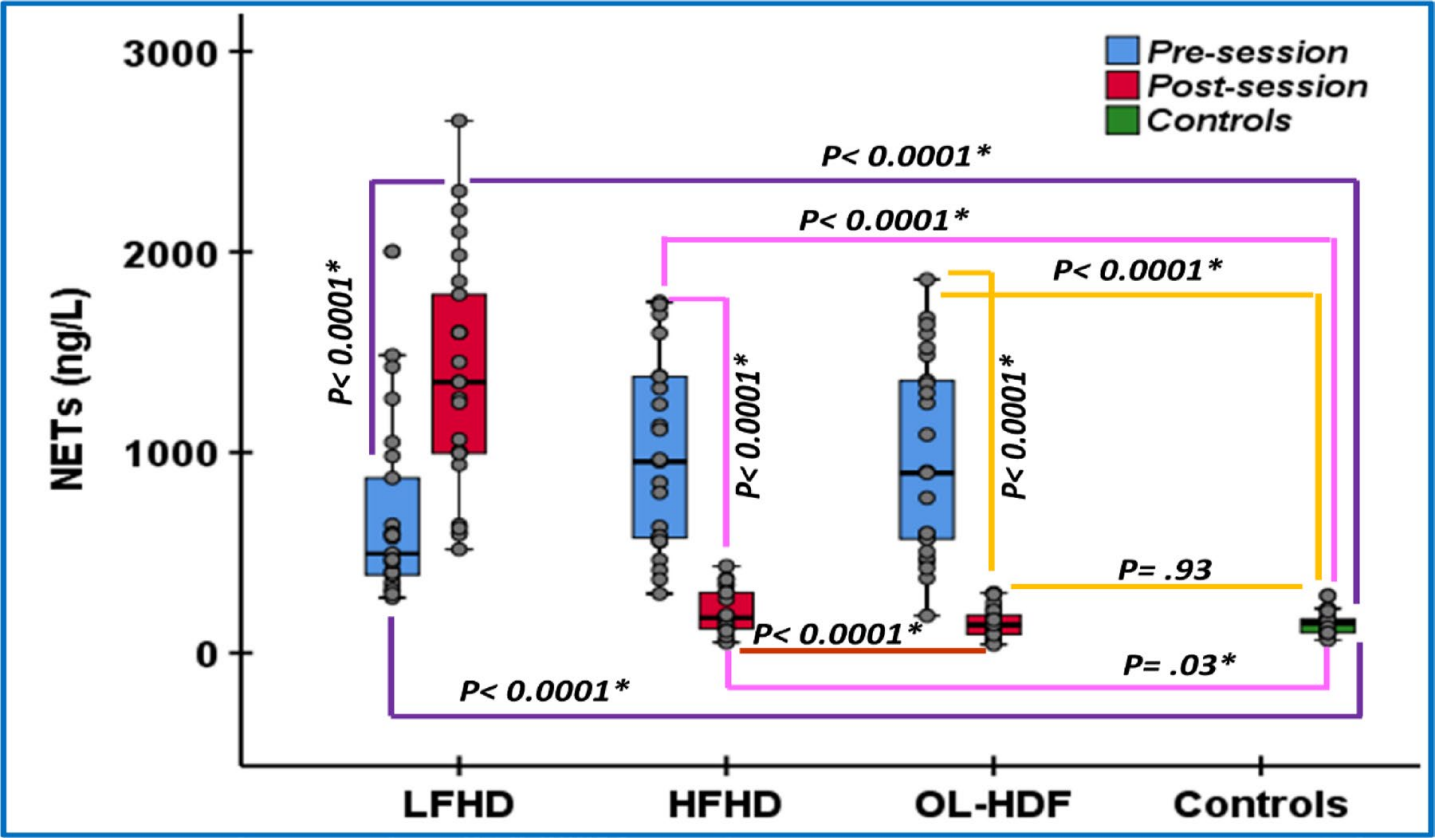
Boxplots of changes in serum NETs’ levels with various HD modalities. NETs, neutrophil extracellular traps; LFHD, low flux hemodialysis, HFHD, high flux hemodialysis; OL-HDF, online hemodiafiltration; ^*^, p-value is statistically significant.

**Fig. 3:**
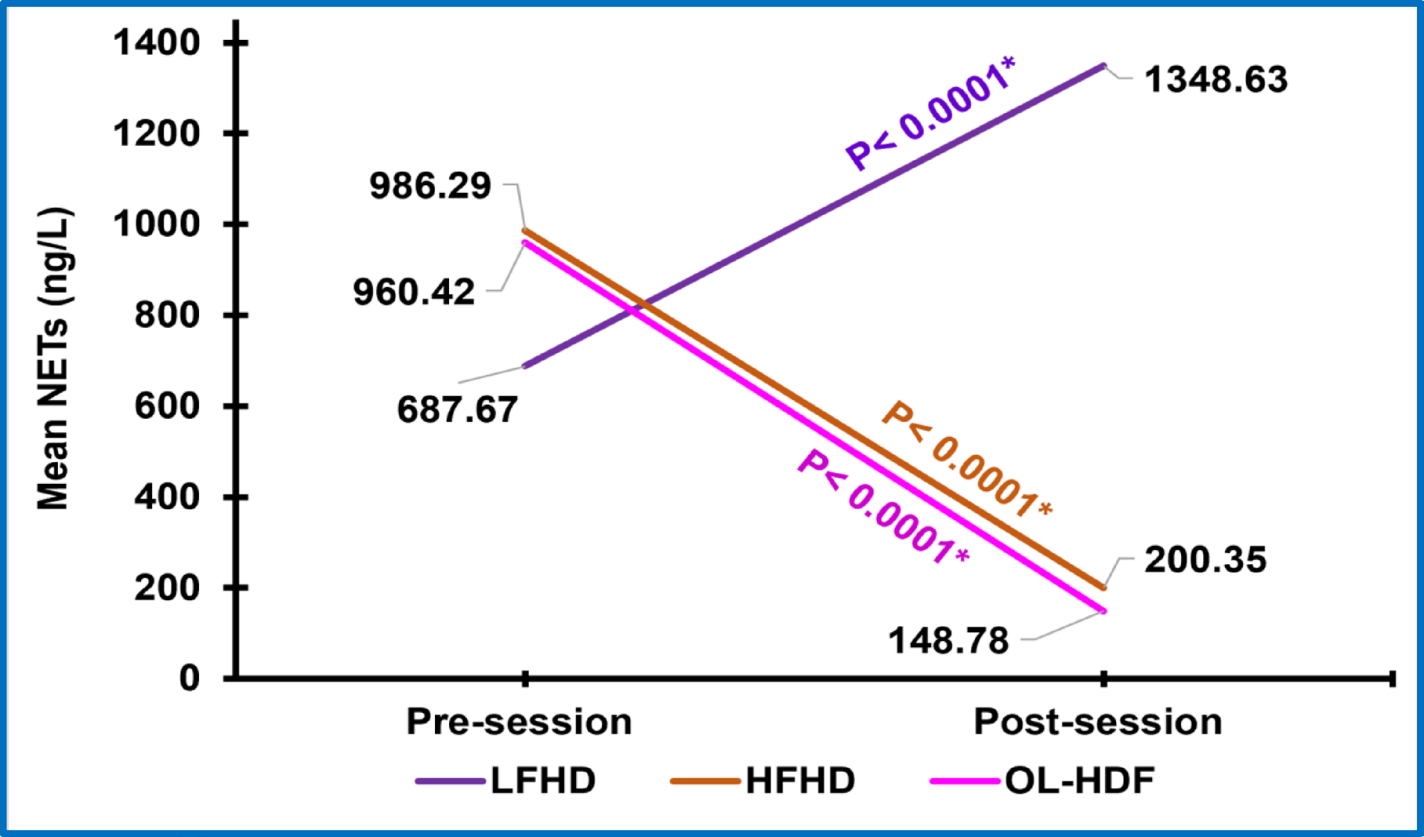
Linear graph showing the trend of mean of changes in serum NETs’ levels with various HD modalities. NETs, neutrophil extracellular traps; LFHD, low flux hemodialysis, HFHD, high flux hemodialysis; OL-HDF, online hemodiafiltration; ^*^, p-value is statistically significant.

Pre-session levels of NETs in LFHD were significantly higher than controls *(p > 0.0001)*. Low Flux HD resulted in a statistically significant further elevation of post-session NETs in comparison to pre-session levels *(p > 0.0001)*. Pre-session NETs’ levels in HFHD and OL-HDF were significantly higher than controls as well *(p < 0.0001 for both)* but were comparable with each other *(p = 0.86)* and to pre-session NETs level in LFHD *(p = 0.05 for both)*. However, both HFHD and OL-HDF resulted in a statistically significant reduction in post-session NETs levels in comparison to pre-session levels (*p < 0.0001 for both*). Post-session NETs levels in OL-HDF were statistically significantly lower than HFHD *(p < 0.0001)*. OL-HDF was the only modality that resulted in a significant reduction of post-session NETs levels to be comparable to that of the control group *(p = 0.93)*. Meanwhile NETs post-session levels in HFHD were still significantly higher than controls *(p = 0.03)*. These results are shown in (Tables 2, 3 and Figs. 2, 3).

**Table 2:** Comparison between serum NETs levels in cases versus controls.

Cases			Controls	*P-value*
Parameters	HD modality	x̄ ± SD *(Mdn ; IQR)*	x̄ ± SD *(Mdn ; IQR)*	
Pre-session NETs (ng/L)	LFHD	687.67 ± 439.90 *(535.95; 519.60)*	147.12 ± 51.73 *(149.05; 69.72)*	*<0.0001* ^a*^
	HFHD	986.29 ± 483 *(954.40; 812.75)*		*< 0.0001* ^b*^
	OL- HDF	960.42 ± 498.55 *(897.60; 883.50)*		*< 0.0001* ^b*^
Post-session NETs (ng/L)	LFHD	1348.63 ± 593.98 *(1310.50; 916.38)*		*< 0.0001* ^b*^
	HFHD	200.35 ± 108.70 *(175.20; 184.69)*		*0.03* ^b*^
	OL- HDF	148.78 ± 76.81 *(139.40; 107.44)*		*0.93* ^*b*^

HD, hemodialysis; x̄ ± SD, mean ± standard deviation; Mdn, median; IQR, interquartile range; NETs, neutrophil extracellular traps; LFHD, low flux hemodialysis, HFHD, high flux hemodialysis; OL-HDF, online hemodiafiltration; ^a^, Mann-Whitney test; ^b^, Student t-test; ^*^, p-value is statistically significant.

**Table 3:** Data and comparisons of NETs values in relation to different HD modalities.

Parameters	HD modality	x̄ ± SD (Mdn; IQR)	*P*-value
Pre-session NETs (ng/L)	LFHD	687.67 ± 439.90 *(535.95; 519.60)*	*0.05* ^*a*^
	HFHD	986.29 ± 483 *(954.40; 812.75)*	
	LFHD	687.67 ± 439.90 *(535.95; 519.60)*	*0.05* ^*a*^
	OL- HDF	960.42 ± 498.55 *(897.60; 883.50)*	
	HFHD	986.29 ± 483 *(954.40; 812.75)*	*0.86* ^*a*^
	OL- HDF	960.42 ± 498.55 *(897.60; 883.50)*	
Post-session NETs (ng/L)	LFHD	1348.63 ± 593.98 *(1310.50; 916.38)*	< 0.0001 ^b*^
	HFHD	200.35 ± 108.70 *(175.20; 184.69)*	
	LFHD	1348.63 ± 593.98 *(1310.50; 916.38)*	< 0.0001 ^b*^
	OL- HDF	148.78 ± 76.81 *(139.40; 107.44)*	
	HFHD	200.35 ± 108.70 *(175.20; 184.69)*	< 0.0001 ^b*^
	OL- HDF	148.78 ± 76.81 *(139.40; 107.44)*	
Pre-vs post-session NETs (ng/L)	LFHD	687.67 ± 439.90 *(535.95; 519.60)*	< 0.0001 ^a*^
	LFHD	1348.63 ± 593.98 *(1310.50; 916.38)*	
	HFHD	986.29 ± 483 *(954.40; 812.75)*	< 0.0001 ^a*^
	HFHD	200.35 ± 108.70 *(175.20; 184.69)*	
	OL- HDF	960.42 ± 498.55 *(897.60; 883.50)*	< 0.0001 ^b*^
	OL- HDF	148.78 ± 76.81 *(139.40; 107.44)*	

HD, hemodialysis; x̄ ± SD, mean ± standard deviation; Mdn, median; IQR, interquartile range; NETs, neutrophil extracellular traps; LFHD, low flux hemodialysis, HFHD, high flux hemodialysis; OL-HDF, online hemodiafiltration; ^a^, Wilcoxon Signed Ranks Test; ^b^, paired t-test; ^*^, p-value is statistically significant.

No significant correlation was found between pre-session NETs’ levels in LFHD and HD vintage *(rho = 0.04*, *p = 0.83)*. Dialyzer surface area in LFHD did not result in any significant difference in post-session NETs’ levels *(p = 0.499).* There were no significant correlations of pump blood flow rate with post-session NETs’ levels in any of the three HD modalities *(rho= -0.13*, *p = 0.53; rho= -0.17*,* p = 0.43; rho = 0.33*,* p = 0.10 for LFHD*,* HFHD and OL-HDF respectively).* There were no significant differences in pre-session NETs’ levels between hypertensive and non-hypertensive patients in LFHD, HFHD and OL-HDF modalities *(p = 0.18*,* p = 0.31*,* p = 0.85 respectively)*. No significant correlations were found between spKt/V with post-session NETs’ levels in any of the 3 HD modalities *(rho= -0.36*, *p = 0.07; rho = 0.18*,* p = 0.40; rho = 0.03*,* p = 0.91 for LFHD*,* HFHD and OL-HDF respectively)*. We did not find any significant correlation between convective volume and post-session NETs’ level in OL-HDF *(r= -0.14*, *p = 0.51)*. Also, there were no significant differences between pre- and post-session counts of total leukocyte count, and absolute neutrophil count in any of the three HD modalities *(p = 0.89*,* p = 0.40; p = 0.73*,* p = 0.47; p = 0.39*,* p = 0.25* for LFHD, HFHD and OL-HDF respectively). Additionally, no significant correlations were found between pre-session NETs’ levels and CRP in LFHD, HFHD and OL-HDF modalities (Table 4 and Supplementary Fig. S1). Neither pre- nor post-session serum NETs’ levels showed any significant correlation with pre- or post-session hemoglobin levels, total leukocyte count, absolute neutrophil count; absolute lymphocyte count, as shown in Table 4 and Supplementary Figs. S2–S9.

**Table 4:** Correlations between NETs levels and hb, TLC, ANC, ALC and CRP.

Timing	HD Modality		CBC Parameters				CRP
			Hb	TLC	ANC	ALC	
Pre-session							
NETs	LFHD	rho	-0.25	0.08	0.03	-0.03	-0.25
		*P-value*	*0.22*	*0.68*	*0.88*	*0.87*	*0.22*
	HFHD	rho	0.08	0.27	0.26	0.04	0.04
		*P-value*	*0.71*	*0.19*	*0.21*	*0.86*	*0.84*
	OL-HDF	rho	-0.11	0.32	0.4	0.10	-0.26
		*P-value*	*0.60*	*0.11*	*0.05*	*0.65*	*0.21*
Post-session							
NETs	LFHD	r	-0.43	-0.17	-0.27	0.04	
		*P-value*	*0.23*	*0.41*	*0.18*	*0.85*	
	HFHD	r	0.32	0.09	0.38	-0.21	
		*P-value*	*0.12*	*0.68*	*0.06*	*0.30*	
	OL-HDF	r	-0.07	0.35	0.31	0.17	
		*P-value*	*0.73*	*0.08*	*0.13*	*0.42*	

HD, hemodialysis; Hb; hemoglobin; TLC, total leukocyte count; ANC, absolute neutrophil count; ALC; absolute lymphocyte count, CRP, C-reactive protein; NETs, neutrophil extracellular traps; LFHD, low flux hemodialysis, HFHD, high flux hemodialysis; OL-HDF, online hemodiafiltration; rho, Spearman’s correlation coefficient; r, Pearson’s correlation coefficient.

While there is extensive research on the immunological implications of maintenance HD, it is still unclear how different HD modalities or various dialyzer functional parameters affect the immune system. Expanding the understanding of these modulations will not only help reduce such inflammatory response but also provide a vital foundation for developing pro-tolerogenic techniques in specific settings, such as HD in septic patients or the final session before kidney transplant in graft candidates. Traditional inflammatory markers like CRP cannot always detect subtle inflammation induced by HD. So, developing new sensitive markers, to evaluate adopting modifications in HD that reduce such immune response, became crucial. NETs levels assay has been postulated as a surrogate for such HD triggered immune response. According to literature review, no research evaluating neutrophil activation in children on HD was undertaken.

Homeostasis of NETs depends on the balance between their formation and degradation. Since the architecture of NETs is made of a meshwork of nuclear and granular proteins attached to a backbone of DNA, the first step in the degradation of NETs is carried on mainly by DNase I. Although DNases destroy the NETs skeleton yet, they cannot remove other NET components that remain adherent to the endothelium. Here comes the role of monocytes/macrophages that engulf NETs and their remnants. This is mostly facilitated by opsonization of NETs by C1q^23^.

The current study demonstrated significantly high basal pre-session levels of circulating NETs in HD patients using polysulfone membranes, whatever the modality or the dialyzer flux used, in comparison to healthy controls. This can be explained by accumulating evidence that chronic uremia triggers a state of uncontrolled NETs production. In vitro studies have shown that the addition of uremic serum increases NET formation, MPO, and nucleosome release from normal neutrophils^23^. They also revealed high baseline NETing and ROS production by neutrophils harvested from the blood of HD patients^23^. Additionally, the partial removal and re-accumulation of uremic toxins due to the intermittent renal replacement therapy that remains as unphysiological treatment, create a chronic state of low-grade inflammation with high rates of oxidative stress affecting all aspects of innate and adaptive immunity^24^. Also, the repeated contact of blood with the dialysis extracorporeal circuit is known to induce a state of low-grade chronic inflammation, neutrophil activation, and oxidative stress^24^.

In our study, LFHD resulted in further elevation of post-session NETs levels, but HFHD and post-dilutional OL-HDF sessions led to significant reduction in post-session NETs levels in comparison to pre-dialysis. Earlier studies investigating the effect of conventional HD on neutrophil activation in different timing related to the dialysis session (pre-, intra-, post) in comparison to healthy controls had discrepant results^23,25–31^. Such heterogeneity can be explained by difference in recruited patients as regards age where most of these studies were conducted in adults, or presence of underlying comorbidities that may influence NETs levels apart from the CKD cases such as diabetes mellitus, cardiovascular events, amyloidosis A, vasculitis, liver transplant, failed kidney graft and on immunosuppression^23,30^.

The major determinant of such diversity across these studies is the different methods used to assess neutrophil activation such as plasma myeloperoxidase, cell-free plasma DNA (cfDNA), NETs (myeloperoxidase [MPO]-DNA complexes), elastase, calprotectin, and cluster of differentiation (CD) markers. Lee et al.^23^ assessed the level of plasma nucleosome (histone-DNA) and myeloperoxidase-DNA in 201 hemodialysis patients and 51 healthy volunteers. Like our study, NETosis markers were significantly higher in HD patients than healthy volunteers. Bieber et al.^30^ assessed NETs level, using myeloperoxidase [MPO]-DNA complexes, before and during HD procedures using polysulfone membranes in 24 adults with kidney failure in comparison to 27 healthy controls. They reported elevated average intradialytic levels of NETs versus pre-dialytic levels. Fukushi et al.^31^ assessed neutrophil activation through measuring plasma levels of MPO before and after HD in 70 adult patients on regular HD with high-performance membrane (polysulfone, 77%; polymethylmethacrylate, 23%). They demonstrated a significant increase in plasma MPO in post-dialysis samples. Costa et al.^27^ reported a statistically significant rise in neutrophil elastase in post-HD versus pre-HD. Aljadi et al.^29^ used the expression of neutrophil activation markers (CD11b, the active epitope of CD11b, and CD88) to measure neutrophil activation in 10 HD patients routinely using high flux polysulfone dialyzers, to be compared to 10 healthy controls. Patients were shifted to a single session using a low-flux polysulfone dialyzer and laboratory assessment was repeated. Peripheral blood samples were drawn before and after HD in each session (one session with high flux and one session with low flux). In contrast to our findings, there were no significant variations in the expression of neutrophil activation markers (CD11b, the active epitope of CD11b, and CD88) when comparing the two different dialyzers before and after dialysis, as well as healthy controls.

Up till now there is no “gold standard” method for detecting NETs in blood or in tissue, and NETosis may be easily evidenced by the detection of surrogate biomarkers, namely NETs byproducts such as circulating cfDNA, citrullinated histone H3 (CitH3), MPO, or elastase^22^. Although ELISA, provides a relatively easy way of NET detection and quantification, yet the scientific community is still in controversy regarding which is the most targeted biomarker for detecting NET formation using this technique. For instance detecting circulating cfDNA does not always indicate NETs since they may be derived from other events, such as apoptosis or necrosis^32^. That may explain the reason why earlier studies^25,26,28^, using cfDNA as a marker for neutrophil activation, showed contradictory results. Also, circulating cfDNA is rapidly degraded in the blood due to the presence of numerous plasma DNases with half- life of 15–20 min. Other ELISA kits detect neutrophil-derived enzymes, such as myeloperoxidase and neutrophil elastase, but these markers may not accurately reflect neutrophil activation, degranulation and NET formation. Recently ELISA for detecting citrullinated histone H3 (CitH3) (the same method employed in our study) was presented and validated for reliable NET quantification with higher specificity in human plasma^33^.

Moreover, other contributing variables that may help us understand such diversity of NETs levels across different studies may include the type of vascular access used in the recruited patients, the site of sampling NETs or at which HD session of the week blood samples were collected. All of these factors may have influenced the results of different studies. We believe that prolonged interdialytic periods are linked to higher interdialytic weight gain and hemodilution which erroneously affect NETs levels. So, we decided to do our laboratory evaluation in a midweek HD session rather than first or last session of the week. Also some of the studies recruited patients who required less than 3 sessions per week, which might reflect that their uremic status was not that severe^26^.

The reason of further elevation of post-session NETs level in LFHD reflects that conventional HD using LF dialyzers has a limited capacity for removal of medium–molecular-weight uremic compounds, suggesting a “residual uremic syndrome” with more production of dialysis induced cytokines and neutrophil activation. The post-session reduction of NETs observed in HFHD and OL-HDF may be attributed to the more efficient clearance of large middle-sized uremic toxins, leading to attenuated production of pro-inflammatory cytokines’ levels —particularly those with short plasma half-lives, such as tumor necrosis factor-alpha (TNF-α), which has an estimated half-life of approximately 15 min. These effects are more pronounced with OL-HDF, which utilizes convective clearance to efficiently remove large middle-molecular-weight solutes, including inflammatory cytokines. So the reduction in systemic inflammation and oxidative stress associated with OL-HDF is likely due to both reduced generation and enhanced clearance of these cytokines^34,35^. Hence, neutrophil activation is expected to be reduced in HFHD and even substantially more reduced in OL-HDF. Similarly Remez-Gabay et al.^36^ suggested that HDF attenuates Netosis due to enhanced biocompatibility and reduced inflammatory responses.

The potential role of hemodialysis-mediated clearance of NETs depends on whether they are present as intact structures or as smaller degradation products. High-resolution scanning electron microscopy (SEM) has shown that NETs consist of fibrillar structures measuring 15–17 nm in diameter, with globular domains of approximately 25 nm that aggregate into threads up to 50 nm^37^.These dimensions exceed the pore sizes of dialysis membranes. Low-flux membranes filter solutes up to ~ 10–15 kDa, while high-flux membranes and online hemodiafiltration extend clearance to ~ 30–60 kDa. However, even these enhanced modalities are incapable of removing intact NETs, whose size and complexity surpass the upper filtration limits of these systems^38^.

In this study, NETs were quantified by measuring citrullinated histone H3 (CitH3), a core structural component of NETs, which may be released into the extracellular space either as a free molecule or bound to DNA within NETs^39^. Regrading the specificity of commercial ELISA kits for NETs based on (CitH3), one may question whether the levels detected exclusively reflect nucleosome-bound (CitH3), or cross reactivity with free (CitH3) may also occur thereby representing both free and nucleosome-bound forms. Based on its small size (~ 15 kDa), free (CitH3) is theoretically dialyzable, even with low flux conventional hemodialysis^40^. However, in our study, post-session NETs level were higher than pre-session levels in patients undergoing LFHD. This paradox can be explained by the fact that free histones, including (CitH3), are highly positively charged and are known to bind to negatively charged plasma proteins, heparins, fibrinogen and platelets, thereby limiting its dialyzability^41^. In addition plasma free (CitH3) is highly unstable and is rapidly degraded by plasma proteases and NET-associated enzymes such as neutrophil elastase and myeloperoxidase suggesting that (CitH3) detected in our assay likely represent the nucleosome-bound form rather than the free fraction^42^. Therefore, post-dialysis reductions in NETs level observed in our study cannot be attributed to clearance via hemodialysis. To confirm such findings measuring (CitH3) in the dialyzate fluid may serve as a valuable approach to validate these obseravations.

The increase in pre-dialysis NETs levels in HFHD and OLHDF can be due to activation of the circulating neutrophils as a result of re-accumulation of uremic toxins because of the intermittent pattern of renal replacement therapy which still remains unphysiological, in addition to the nature of high baseline NETing of HD patients’ neutrophils.

Bieber et al.^30^ suggested that the role of other undetermined variables in neutrophil activation during HD procedure such as blood and dialysate flow rates, dialysate composition, dialyzer surface area, tubing structure and composition, and the length of HD session. In our study, we did not find any statistically significant difference in post-HD NETs level in relation to dialyzer surface area and blood flow rate. This finding should be cautiously considered because of small sample size and limited variability in dialyzer surface area and blood flow rate.

According to Fukushi et al.^31^ TLC, neutrophils, ANC, LC and monocyte count decreased significantly with HD along with significant increases of the early apoptotic ratio of WBCs, neutrophils and monocytes after HD, but not lymphocytes. These increases did not show any significant correlation with cellular counts. The later finding agrees with our results, since we did not find any significant correlation between pre- and post-HD NETs level and TLC, ANC, and ALC implicating that it is a matter of alteration in leukocyte function rather than number.

We acknowledge that the limitations of this study include small sample size, lack of randomization, and short duration of HFHD and OL-HDF treatments. Hence our assessment may not reflect the real impact of these modalities on pre-session NETs level. Additionally, a cross over study design would have been more preferred, however it was not applicable due to feasibility, and cost issues. Also, LFHD is not a preferred method of HD anymore in many dialysis centers, yet sometimes the availability of dialyzers, or rather unavailability, dictates the use of this kind of dialysis even for limited periods of time especially in developing countries. Lastly, an important limitation is the lack of measurement of NETs in the dialysate fluid, which could have provided additional confirmation and greater insight into the observed variations in NETs level across different HD modalities.

## Conclusion

The current study showed that children on HD have high serum basal levels of NETs that significantly increased after LFHD. HFHD and OL-HDF led to significant reduction in NETs levels reflecting their effective role in removing large middle-sized uremic toxins and reducing pro-inflammatory cytokines with less dialysis induced inflammation leading to reduction of dialysis related production of NETs. However, OL-HDF was superior to HFHD, as it lowered post-session NETs to levels comparable to controls.

## Supplementary Information

Below is the link to the electronic supplementary material.

Supplementary Material 1

## Data Availability

Data will be available upon request to the corresponding author.
