# Supplementary material for: Differential effects of hemodialysis modalities on circulating neutrophil extracellular traps in children on maintenance hemodialysis: a cohort study
Source: Sci Rep. 2025 Sep 17;15:32575. doi: 10.1038/s41598-025-18791-4 (PMC12443955; doi:10.1038/s41598-025-18791-4)
Supplement: Supplementary file 1 — Supplementary Material 1 [file 41598_2025_18791_MOESM1_ESM.docx]

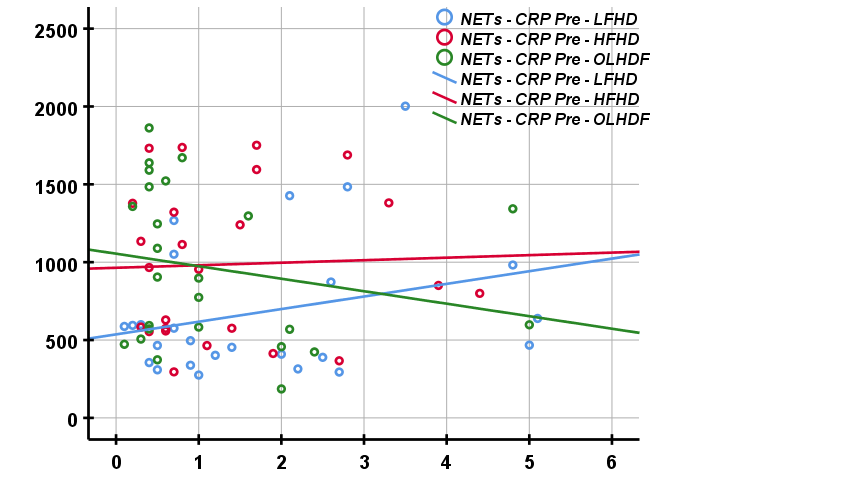


**NETs (ng/L)**

***R^2^ = 0.080; p= 0.22***

***R^2^ = 0.002; p= 0.84***

**CRP mg/L**

***R^2^ = 0.044; p= 0.21***

**NETs (ng/L)**

***R^2^ = 0.046; p= 0.30***

**ALC x 10^9^/L**

***R^2^ = 0.028; p= 0.42***

**NETs (ng/L)**

***R^2^ = 0.046; p= 0.30***

***R^2^ = 0.028; p= 0.42***

**Supplementary Fig. S1*Correlation between pre-session neutrophil extracellular trap (NET) levels and CRP (C - reactive protein) in patients undergoing low-flux hemodialysis (LFHD), high-flux hemodialysis (HFHD), and online hemodiafiltration (OLHDF). Linear regression with (R²) and p-values are shown for each modality.***


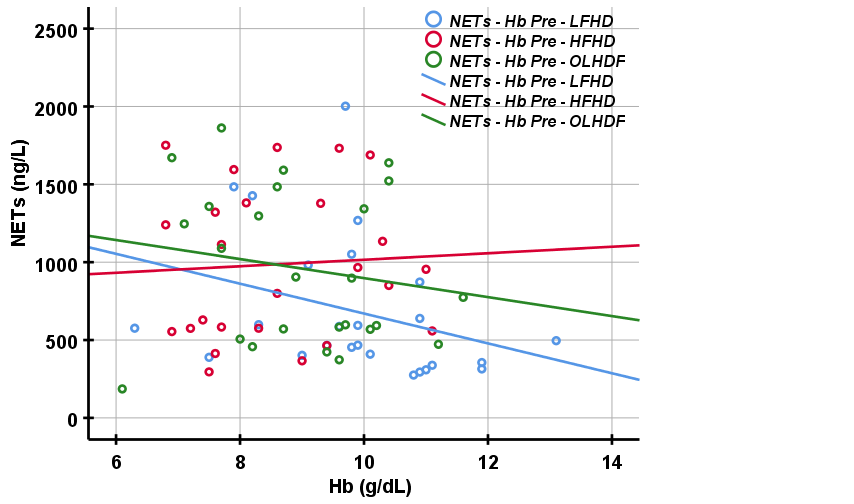


***R^2^ = 0.003; p= 0.71***

***R^2^ = 0.03; p= 0.60***

***R^2^ = 0.09; p= 0.22***

**Supplementary** **Fig.S2: *Correlation between pre-session neutrophil extracellular trap (NET) levels and hemoglobin (Hb) levels in patients undergoing low-flux hemodialysis (LFHD), high-flux hemodialysis (HFHD), and online hemodiafiltration (OLHDF). Linear regression with (R²) and p-values are shown for each modality.***


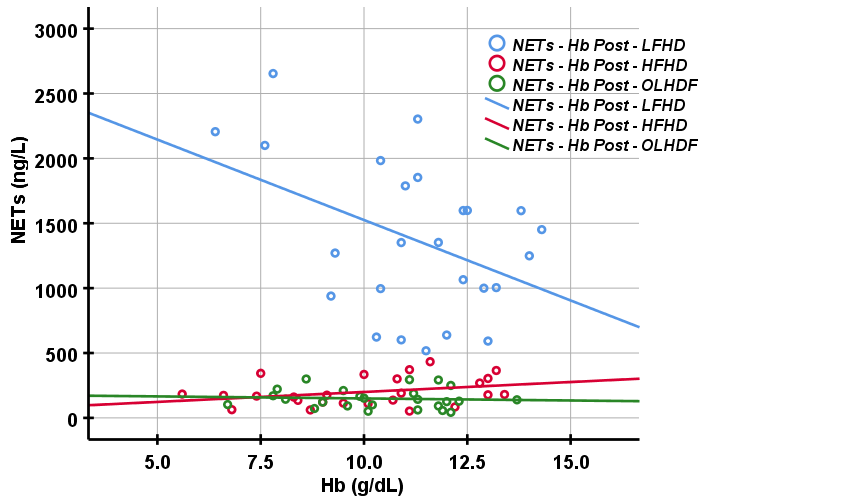


***R^2^ = 0.102; p= 0.12***

***R^2^ = 0.005; p= 0.73***

***R^2^ = 0.102; p= 0.23***

**Supplementary** **Fig.S3:** ***Correlation between post-session neutrophil extracellular trap (NET) levels and hemoglobin (Hb) levels in patients undergoing low-flux hemodialysis (LFHD), high-flux hemodialysis (HFHD), and online hemodiafiltration (OLHDF). Linear regression with (R²) and p-values are shown for each modality.***


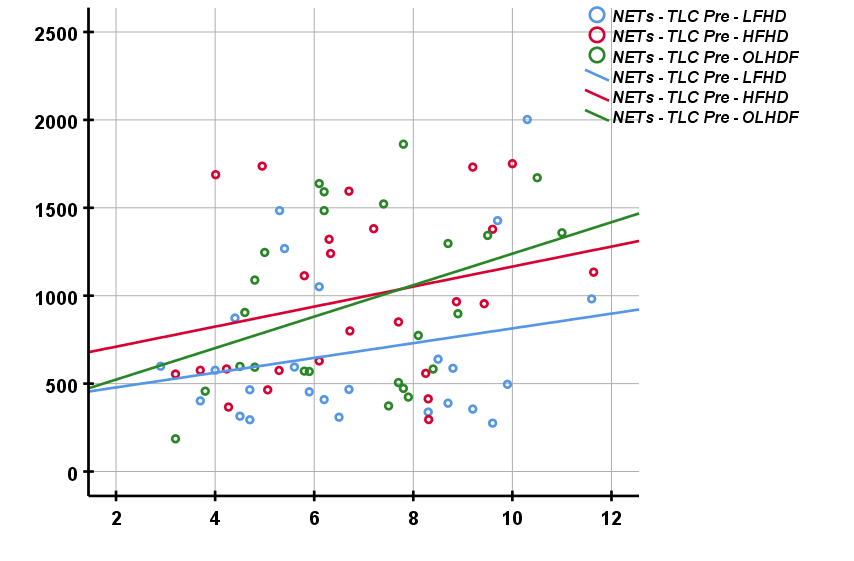


**NETs (ng/L)**

**TLC x 10^9^/L**

***R^2^ = 0.045; p= 0.68***

***R^2^ = 0.069; p= 0.19***

***R^2^ = 0.137; p= 0.11***

**Supplementary** **Fig.S4: *Correlation between pre-session neutrophil extracellular trap (NET) levels and TLC (Total Leukocyte Count) in patients undergoing low-flux hemodialysis (LFHD), high-flux hemodialysis (HFHD), and online hemodiafiltration (OLHDF). Linear regression with (R²) and p-values are shown for each modality.***


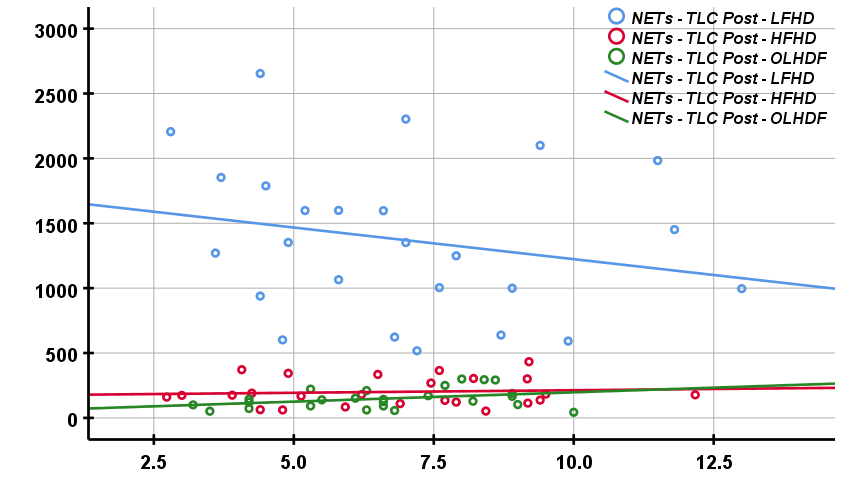


**NETs (ng/L)**

**TLC x 10^9^/L**

***R^2^ = 0.028; p= 0.41***

***R^2^ = 0.008; p= 0.68***

***R^2^ = 0.124; p= 0.08***

**Supplementary** **Fig.S5: *Correlation between post-session neutrophil extracellular trap (NET) levels and TLC (Total Leukocyte Count) in patients undergoing low-flux hemodialysis (LFHD), high-flux hemodialysis (HFHD), and online hemodiafiltration (OLHDF). Linear regression with (R²) and p-values are shown for each modality.***


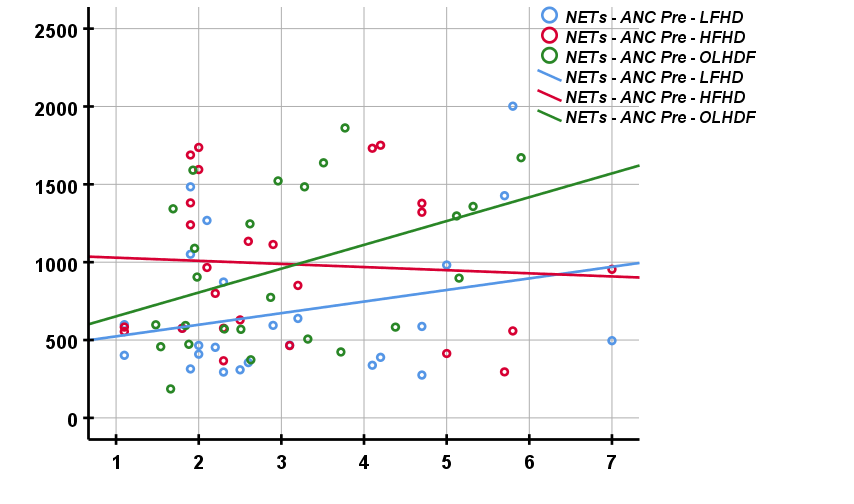


**NETs (ng/L)**

***R^2^ = 0.061; p= 0.88***

***R^2^ = 0.095; p= 0.21***

**ANC x 10^9^/L**

***R^2^ = 0.162; p= 0.05***

**Supplementary** **Fig.S6: *Correlation between pre-session neutrophil extracellular trap (NET) levels and ANC (Absolute Neutrophil Count) in patients undergoing low-flux hemodialysis (LFHD), high-flux hemodialysis (HFHD), and online hemodiafiltration (OLHDF). Linear regression with (R²) and p-values are shown for each modality.***


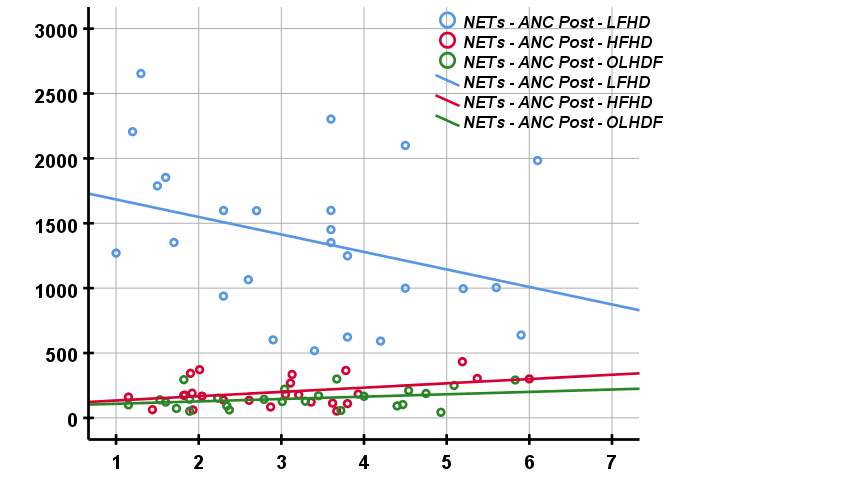


**NETs (ng/L)**

***R^2^ = 0.075; p= 0.18***

***R^2^ = 0.144; p= 0.06***

**ANC x 10^9^/L**

***R^2^ = 0.099; p= 0.13***

**Supplementary** **Fig.S7: *Correlation between Post-session neutrophil extracellular trap (NET) levels and ANC (Absolute Neutrophil Count) in patients undergoing low-flux hemodialysis (LFHD), high-flux hemodialysis (HFHD), and online hemodiafiltration (OLHDF). Linear regression with (R²) and p-values are shown for each modality***


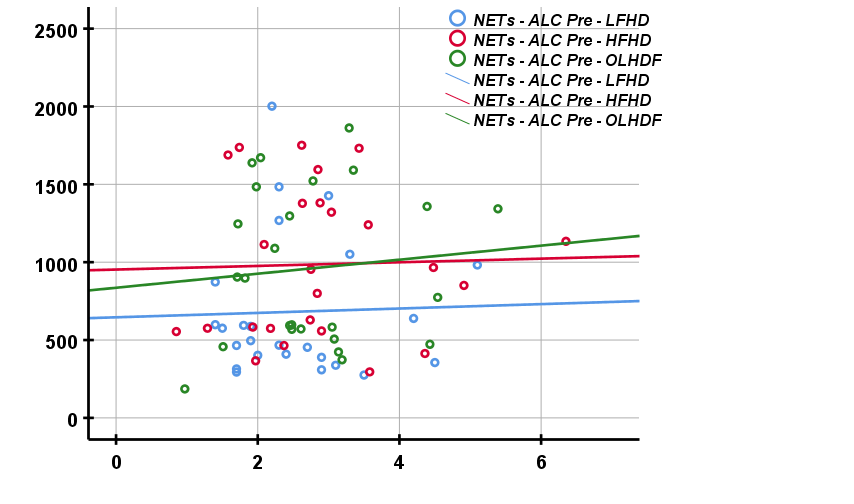


**NETs (ng/L)**

***R^2^ = 0.001; p= 0.87***

***R^2^ = 0.001; p= 0.86***

**ALC x 10^9^/L**

***R^2^ = 0.009; p= 0.65***

**Supplementary** **Fig.S8: *Correlation between pre-session neutrophil extracellular trap (NET) levels and ALC (Absolute Lymphocyte Count) in patients undergoing low-flux hemodialysis (LFHD), high-flux hemodialysis (HFHD), and online hemodiafiltration (OLHDF). Linear regression with (R²) and p-values are shown for each modality.***


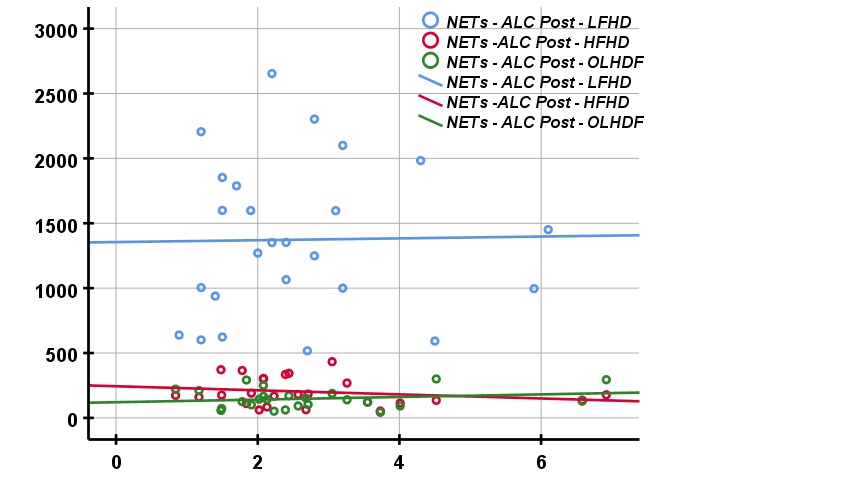


**NETs (ng/L)**

***R^2^ = 0.002; p= 0.85***

***R^2^ = 0.046; p= 0.30***

**ALC x 10^9^/L**

***R^2^ = 0.028; p= 0.42***

**Supplementary** **Fig.S9: *Correlation between post-session neutrophil extracellular trap (NET) levels and ALC (Absolute Lymphocyte Count) in patients undergoing low-flux hemodialysis (LFHD), high-flux hemodialysis (HFHD), and online hemodiafiltration (OLHDF). Linear regression with (R²) and p-values are shown for each modality.***
